# Multi-Domain Airflow Modeling and Ventilation Characterization Using Mobile Robots, Stationary Sensors and Machine Learning

**DOI:** 10.3390/s19051119

**Published:** 2019-03-05

**Authors:** Victor Hernandez Bennetts, Kamarulzaman Kamarudin, Thomas Wiedemann, Tomasz Piotr Kucner, Sai Lokesh Somisetty, Achim J. Lilienthal

**Affiliations:** 1 Mobile Robotics and Olfaction Lab, Örebro University, 702 81 Örebro, Sweden; Tomasz.Kucner@oru.se (T.P.K.); achim.lilienthal@oru.se (A.J.L.); 2 Center of Excellence for Advanced Sensor Technology, School of Mechatronics Engineering, Universiti Malaysia Perlis, Arau Perlis 02600, Malaysia; kamarulzaman@unimap.edu.my; 3 Institute of Communications and Navigation, German Aerospace Center, 82234 Oberpfaffenhofen, Germany; Thomas.Wiedemann@dlr.de; 4 Department of Mechatronics, Sastra University, Thanjavur 613401, India; slvslokesh@gmail.com

**Keywords:** airflow modeling, ventilation, mobile robotics, static sensor networks, environmental monitoring, machine learning

## Abstract

Ventilation systems are critically important components of many public buildings and workspaces. Proper ventilation is often crucial for preventing accidents, such as explosions in mines and avoiding health issues, for example, through long-term exposure to harmful respirable matter. Validation and maintenance of ventilation systems is thus of key interest for plant operators and authorities. However, methods for ventilation characterization, which allow us to monitor whether the ventilation system in place works as desired, hardly exist. This article addresses the critical challenge of ventilation characterization—measuring and modelling air flow at micro-scales—that is, creating a high-resolution model of wind speed and direction from airflow measurements. Models of the near-surface micro-scale flow fields are not only useful for ventilation characterization, but they also provide critical information for planning energy-efficient paths for aerial robots and many applications in mobile robot olfaction. In this article we propose a heterogeneous measurement system composed of static, continuously sampling sensing nodes, complemented by localized measurements, collected during occasional sensing missions with a mobile robot. We introduce a novel, data-driven, multi-domain airflow modelling algorithm that estimates (1) fields of posterior distributions over wind direction and speed (“ventilation maps”, spatial domain); (2) sets of ventilation calendars that capture the evolution of important airflow characteristics at measurement positions (temporal domain); and (3) a frequency domain analysis that can reveal periodic changes of airflow in the environment. The ventilation map and the ventilation calendars make use of an improved estimation pipeline that incorporates a wind sensor model and a transition model to better filter out sporadic, noisy airflow changes. These sudden changes may originate from turbulence or irregular activity in the surveyed environment and can, therefore, disturb modelling of the relevant airflow patterns. We tested the proposed multi-domain airflow modelling approach with simulated data and with experiments in a semi-controlled environment and present results that verify the accuracy of our approach and its sensitivity to different turbulence levels and other disturbances. Finally, we deployed the proposed system in two different real-world industrial environments (foundry halls) with different ventilation regimes for three weeks during full operation. Since airflow ground truth cannot be obtained, we present a qualitative discussion of the generated airflow models with plant operators, who concluded that the computed models accurately depicted the expected airflow patterns and are useful to understand how pollutants spread in the work environment. This analysis may then provide the basis for decisions about corrective actions to avoid long-term exposure of workers to harmful respirable matter.

## 1. Introduction

Ventilation systems are a central component of most buildings, especially in public and work spaces. In heavy industry, for example in foundries or mines, proper ventilation is often crucial for health and safety of the workers. For instance, efficiently providing all parts of a mine in which humans operate with sufficient air is essential and a significant cost factor for running a mine. Ventilation systems that do not work properly can cause severe accidents. An example is the Upper Big Branch mine disaster that occurred on 5 April 2010, in Raleigh County, West Virginia, USA (https://en.wikipedia.org/wiki/Upper_Big_Branch_Mine_disaster). An inquiry by the Department of Labour (DOL) found that improper ventilation contributed to the accident and, as a preventive action, the DOL urged mining operators to enforce ventilation regulations strictly. Moreover, the DOL specifically stated that intentional changes in the ventilation systems, such as the addition of new fans and shafts, can alter the airflow direction in a way that mine safety is compromised. Ventilation systems also play an essential role in avoiding health issues through long-term exposure to harmful respirable matter. By diluting and displacing indoor pollutants, ventilation systems in foundries and similar work environments are designed to reduce workers’ exposure to, e.g., particulate matter that can cause irreversible health disorders [1].

It is thus evident that not only the design but also validation and maintenance of ventilation systems is of key interest for plant operators and authorities. However, ventilation surveying techniques, which capture the complexities of indoor ventilation systems, hardly exist. Thus, methods for ventilation characterization need to be developed that allow to monitor whether the ventilation system in place works as desired. Existing data-driven commercial techniques do not allow for dense spatial and temporal modelling of the variability of indoor airflow since they use only sporadic measurements, taken at few locations, to compute performance metrics. Alternatives based on Computational Fluid Dynamics can produce sophisticated ventilation models. However, their accuracy is highly dependent upon the precise specification of the boundary conditions, which are typically unavailable at the desired accuracy. Additionally, these models suffer from errors in the flow domain geometry arising from modifications to the structures through partitions and furnishings.

**Figure 1:**
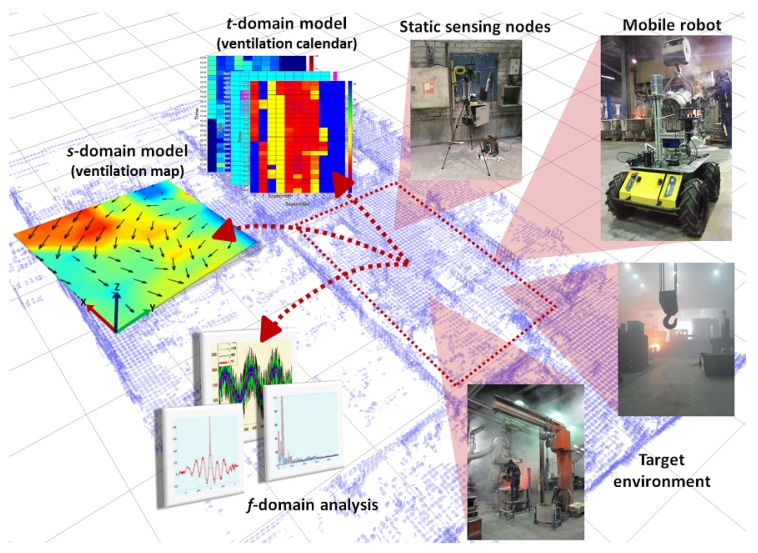
Block diagram of the proposed ventilation characterization system. Data from mobile robots and sensing nodes are combined to produce temporal, spatial and frequency domain models.

The work presented in this paper is summarized in Figure 1. We combine localized measurements collected with a surveying mobile robot and data collected with static sensing nodes to perform ventilation characterization. We propose the stf-AFM data-driven algorithm that performs airflow modeling (AFM) in three different domains, namely the spatial (s), temporal (t) and frequency (*f*) domain. The s model combines the airflow observations into a spatial ventilation map, which shows flow (and its variability) at different locations of the surveyed area. The t model computes ventilation calendars, which show the airflow variability in the form of time-limited (e.g., hourly) probability density functions. Both, ventilation map and ventilation calendars capture the airflow variability, and the suggested approach incorporates the sensor model, which are, to the best our knowledge, novel contributions compared to state-of-the-art airflow modeling algorithms. The f model results from a frequency domain analysis to identify frequency bands corresponding to dominating periodic changes of airflow in the environment (e.g., changes in the wind direction).

The stf-algorithm follows in spirit the key sensing principle behind the hybrid pollution monitoring system presented in [1]. Since monitoring robots could not be guaranteed 24/7 access to the industrial premises, a hybrid sensing approach, comprising of a stationary sensor network and an occasionally present mobile robot was chosen. In this way, spatially-dense but temporally sparse models can be obtained with the mobile robot (whenever possible) and can be used to complement the temporally-dense but spatially sparse data acquired with the on-site sensor network. Notably, the advantage of such a robot-aided monitoring systems is not only that the robots can learn dense pollution/wind models at adaptively chosen locations. Their role is also to learn spatial extrapolation models, which are then used to obtain dense concentration fields from the stationary sensor network measurements [2].

We tested our approach first with simulated data and with experiments in a semi-controlled environment. In this way, we verified the accuracy of the proposed approach (in comparison to traditional airflow modeling algorithms), and its sensitivity to different noise levels. We considered specifically noise in the form of turbulence and short-term airflow changes induced by activities and movement nearby the wind sensors. In addition, we deployed our system (Figure 1) in two different industrial environments in full operation and with different ventilation regimes. The system operated for a total of three weeks where disruptions from daily work activities were present. We presented the generated models to plant operators to perform a qualitative evaluation of the system. The plant operators concluded that the computed models accurately depicted the expected airflow patterns and moreover, such models are useful to understand how pollutants spread in the work environment. Such an analysis may then provide the basis for decisions about corrective actions to avoid long-term exposure of workers to harmful respirable matter.

We would like to point out that the basic principles of robot-aided sensor network approaches also apply to the field of urban pollution monitoring. Authorities could use mobile sensing units to improve air pollution models based on data from stationary weather stations, which are usually sparse in number. These models can in turn help city planners to understand how different attributes of the urban form interact and affect pollution levels. An example where measurements obtained on public buses (i.e., in comparison to this paper, with less control over the measurement locations) were used together with samples from traditional urban measurement stations is the work of Marjovi et al. [3].

The results presented in this paper were chiefly developed during the research projects SURVEYOR (https://mro.oru.se/projects/surveyor/) (surveying ventilation systems in foundries using robots and stationary sensors, Vinnova Sip STRIM Project Nr. 2017-05468, 2018). Accordingly, the validation experiments presented in this article were carried out in foundry halls. We would like to reiterate, however, that our results are of course not limited to the casting business sector. We have received already signals of interest for improved ventilation characterization systems from the smart buildings sector, mining, automotive manufacturing and other sensitive work environments.

The contributions of this paper can be summarized as follows:

- We propose a data-driven approach to obtain a multi-domain (spatial/temporal/frequency) view of indoor airflow in general, which is useful for ventilation characterization in particular. This is relevant for, e.g., environmental monitoring robots and occupational health inspections. Data-driven airflow modeling is not trivial due to the complexities of turbulence. To the authors’ best knowledge, no other state-of-the-art, data-driven modeling techniques offer such a comprehensive view of the airflow phenomena.
- We expand conventional micro-scale airflow density estimation techniques to consider sensors characteristics. Introducing a sensor model in the computations allows to account for the uncertainties induced by the sensors’ limitations.
- We propose to fuse process models in the computation of the airflow density models. The introduction of the process models allows the PDF estimation process to discriminate between dominant, laminar airflow and short term variations due to turbulence.
- We develop a frequency analysis method to study periodic changes in wind direction. Due to the circular nature of wind direction, conventional correlogram-based techniques are not directly applicable. We thus developed a technique that combines circular correlation, the fast Fourier transform and mean shift clustering to identify the periodicity of events that have a high impact on airflow direction changes.
- We conduct quantitative evaluation of the proposed airflow density estimation approach. We use simulated data (from a conventional airflow model) and as well as data acquired in semi-controlled environments. This due to the fact that, in complex environments such as foundries, it is not feasible to acquire ground truth data since different factors, such as temperature gradients and work activity, influence airflow.
- We deploy and perform qualitative analysis of the proposed heterogeneous wind estimation system in two foundry sites, which have different ventilation regimes. These deployments produced a series of models that were evaluated with the help of occupational health specialist of the respective foundries. The input from the specialists demonstrated that the models correlated with the expected airflow conditions and as well allowed to identify periodic activities in the foundry that have an important impact on airflow changes.

This article is structured as follows, in Section 2, we present related work on ventilation characterization and AFM in micro scales. Section 3 presents the multi-domain AFM we propose as a major contribution in this article. Section 5 describes the hybrid monitoring system (mobile robot + sensing nodes) developed for the purpose of ventilation characterization in industrial environments. Quantitative validation conducted with simulations and semi-controlled experiments is presented in Section 4. The results from our system deployment in two different foundries are presented in Section 6. A discussion, conclusions and final remarks conclude the paper in Section 7.

## 2. Related Work

Ventilation characterization, the process of measuring, modelling and evaluating the effects of ventilation systems, is of great interest for plant operators and governmental authorities. Ventilation characterization is challenging primarily due to the complexity of indoor airflow, which is typically dominated by turbulence and thus is characterized by eddies and vortices at different scales as well as unstable and changing patterns at different locations in the work space. A ventilation characterization process that accurately models indoor airflow has yet to be fully realized.

Methods for ventilation characterization aim to build a model of the actual spatio-temporal airflow patterns in a given environment and to compare it to the airflow pattern intended by the designer. The key aspect we focus on in this article is micro-scale (<2 km) air flow modelling (μAFM), that is, creating a high-resolution model of wind speed and direction from airflow measurements in the atmospheric boundary layer. Near surface micro-scale flow fields are critical information for aerial robots, especially in planning energy-efficient paths in heterogeneous wind fields [4]. The μAFM models are also important for applications in mobile robot olfaction (MRO). Here, wind data can be used to improve spatial modelling of gas distributions [5] or to determine exploration trajectories of gas-sensitive robots [6,7]. It is also promising to integrate airflow models into gas source localization approaches, but this has not been done so far to the best of our knowledge.

Common ventilation characterization methods include the use of tracers and the sporadic collection of measurements. Also, ventilation systems can also be evaluated by estimating the changes in worker’s exposure levels using data collected with different pollution sensors [8]. While these approaches are relatively easy to implement, they do not capture the spatial variability of the ventilation systems and, moreover, they do not provide any estimation about the direction or the speed of the airflow, which is a critical variable, for example inside mines, where the accumulation of flammable gases can have serious consequences. Alternative approaches based on computational fluid dynamics (CFD) techniques can produce sophisticated ventilation models [9]. However, their accuracy relies on precise knowledge about boundary conditions, which is often not available.

The μAFM can be carried out by combining CFD, and data-driven techniques in a method referred to as spatial downscaling [10]. The measured data acquired at sparse measurement stations is downscaled by CFD models that consider the topography and ambient conditions (e.g., estimated temperature fields) of the area of interest.

AFM approaches that rely only on data-driven, statistical methods have also been proposed, such as in [11], where an algorithm that uses large historical datasets to infer small-scale models (appropriately 0.5 km resolution) was presented. Long-term data-driven AFM algorithms for scales below 0.5 km, or indoor AFM have yet to be realized. Most of the data-driven AFM approaches for scales below 0.5 km create time-limited snapshots of the airflow distribution that disregard the direction of the wind flow [12,13].

In the presented work, we not only model the air flow in the spatial and temporal domain but also investigate air flow (in particular changes in the wind direction) in the frequency domain. In our work, we combine correlograms, the Fourier transform and clustering methods to detect periodicities in the collected measurements. A similar problem has been investigated by Krajnik et al. [14]. The authors introduce FreMen, a spectral model that represents the uncertainty of the estimated state variables. The key difference between FreMen and the frequency domain models presented in this paper is that FreMen is tailored to model binary variables. In contrast, in this work, we are focusing on building frequency models of wind direction, which is a continuous, circular variable. Furthermore, in state-of-the-art work, the analysis of airflow patterns has not been fully explored. Examples of frequency domain analysis for airflow data include the work of Syōno and Tanaka [15]. The authors build their work on the much earlier contribution of Ertel [16]. In this work, the authors decompose the curves followed by wind speed changes and wind directions changes using Fourier transform in this way obtaining a reliable way to predict the concentration of future measurements.

## 3. The *stf*-AFM Algorithm

Several related works address the problem of modeling the wind flow at macroscopic scales. However, only a few publications address the problem of airflow mapping at micro-scales focusing on the probabilistic estimation of airflow fields [11,12,13]. In this section we present the *stf*-AFM Algorithm, which extends the state-of-the-art and allows to create airflow models on spatial (*s*), temporal (*t*) and frequency (*t*) domains. The *s*- and *t*-domains of our algorithm rely on the estimation of probability density function (PDF)s. First, we improve the mixture models introduced in [17,18] by introducing the sensor and process models in the computation of the PDF. These improvements allow to filter out noisy, sudden airflow changes that can be produced by turbulence or activity in the surveyed environment. Due to this feature, our sensor-aware PDF estimation algorithm allows to identify dominant airflow streams.

As previously stated, the sensor-aware PDF estimation algorithm is used in the *s*- and *t*-models. In the case of the *t*-model, we compute time-limited PDFs. This means that a PDF is computed from data collected during, e.g., one hour, one work day, or two work weeks. The variability between subsequent time-limited PDFs of the airflow is captured in a process or transition model [19] that is learned from the data for each time interval in the model. We refer to the *t* domain models as ventilation calendars.

The *s*-domain model is computed using the LT algorithm, originally introduced in [19]. The LT algorithm creates a representation in which airflow is modeled as a combination of laminar and turbulent components (analogous to conventional airflow modeling approaches, as explained in [20,21]). The model is created from localized measurements at different locations combined with an extrapolation algorithm tailored for airflow estimation that tries to estimate laminar and turbulent components and predicts PDFs at arbitrary query locations. Examples of ventilation maps and ventilation calendars are shown in Section 6.

We compute an *f*-domain representation with a novel algorithm, which allows to identify dominating frequency bands of likely temporal, periodic patterns. The novel sensor-aware PDF modeling algorithm, the LT algorithm and the novel *f*-domain modeling algorithm are described in the following subsections.

### 3.1. Sensor-Aware PDF Estimation

Common approaches to estimating PDF consider wind speed and wind direction as independent variables and estimate the parameters or parametric distributions, e.g., Weibull (wind speed) and Von–Mises (wind direction), from a series of measurements. However, unimodal distributions do not offer sufficient expressiveness to capture the complexities of, e.g., turbulent flow where multi-modal distributions are typical. A solution to this problem, which is also adopted in our approach, is to consider mixture models instead of unimodal distributions. Existing approaches make the implicit assumption of an ideal airflow sensor, which means that the sensor outputs exactly the same value every time if exposed to the same true airflow vector. To incorporate the specific sensor characteristics into the task of airflow modeling, we presented an approach to estimate a model-free probability mass function (PMF) that relies on a sensor model and also learns the process model (i.e., the wind flow variability) from measurements [19]. This PMF modeling algorithm is based on a probabilistic filter and models the joint PMF (wind speed and direction) as a discrete, two dimensional space $\Gamma_{\theta ,\nu }$ that has a total of *N* states, with *N* given by the discretization parameters. Each state in $\Gamma_{\theta ,\nu }$ is a (θ,ν) tuple of wind direction and speed, computed from a set of measurements z=(θ,ν) taking into account a sensor model $N_{s}\left(z|\hat{\sigma_{\theta }},\hat{\sigma_{\nu }}\right)$, with the confidence parameters $\hat{\sigma_{\theta }}$ and $\hat{\sigma_{\nu }}$ of the sensor, which are typically given by the manufacturer. We do not make an a priori assumption about the process model and instead, chose a frequentist approach. The main idea behind learning the process model is to identify the variability associated to different components of the wind flow model, which then allows us to assign higher posterior probabilities to components that do not exhibit frequent, intermittent transitions. This allows, in an online fashion, to discriminate between those rather stable components, which lend themselves to extrapolation, and residual, strongly varying components akin to turbulent fluctuations. Further implementation details can be found in [19].

In this work, we further developed the work presented in [19] and the work of [17,18] to estimate sensor-aware PDFs based on mixture models. These mixture models comprise Von–Mises and Weibull distributions which, as previously stated, are a standard choice in different wind-related applications [22]. In the following two subsections, we first introduce the mixture models and their parameters. Then, we explain how to optimize the model parameters while incorporating the sensor model.

#### 3.1.1. The Von Mises Mixture Model

As described in [17], a mixture of Von–Mises distributions (denoted by mvM(θ)) is a continuous model used to characterize wind direction. mvM(θ) is constructed as a sum of *N* Von–Mises probability density functions as follows:

(1) $$mvM\left(\theta \right)=\sum_{j=1}^{N}\frac{\omega_{j}}{2\pi I_{0}\left(\kappa_{j}\right)}exp\left[\kappa_{j}cos\left(\theta -\mu_{j}\right)\right]$$

In the previous equation, $\omega_{j}$ are non-negative mixing weights with $0\leq \omega_{j}\leq 1$ and $\sum_{j}\omega_{j}=1$. $\mu_{j}$ is the mean direction while $\kappa_{j}$ is referred to as the concentration parameter. $I_{0}\left(\kappa_{j}\right)$ corresponds to the Bessel function of the first kind and order zero [23]. As can be seen in Equation (1), the Von–Mises mixture model (mvM) depends on the parameters ω, μ, *N* and κ.

#### 3.1.2. The Bi-Modal Weibull Model

The work presented in [18] proposes to use a bi-modal Weibull model to represent heterogeneous wind speed regimes. Indeed, the complexities of indoor airflow can be better captured by a mixture model. A narrow Weibull distribution, for example, may be used to model nearly constant low-speed measurements while a wide Weibull distribution can be used to model changing high-speed wind (as can be seen in the models shown in Section 6). In [18] the authors define the bi-modal Weibull distribution as follows:

(2) $$bWB\left(\nu \right)=\omega \cdot \left\{\frac{\alpha_{1}}{\beta_{1}}{\left(\frac{\nu }{\beta_{1}}\right)}^{\alpha_{1}-1}exp\left[-{\left(\frac{\nu }{\beta_{1}}\right)}^{\alpha_{1}}\right]\right\}+\left(1-\omega \right)\cdot \left\{\frac{\alpha_{2}}{\beta_{2}}{\left(\frac{\nu }{\beta_{2}}\right)}^{\alpha_{2}-1}exp\left[-{\left(\frac{\nu }{\beta_{2}}\right)}^{\alpha_{2}}\right]\right\}.$$

In the above equation, 0≤ω≤1 is the mixing factor, and the wind speed sample is denoted by ν while α and β are shape and scale parameters respectively.

#### 3.1.3. Mixture Model Parameter Estimation

In [17,18], a least square approach is proposed to estimate the parameters for the mixture models bWB and mvM as the following non linear programming problem:

(3) $$\begin{array}{cc} \underset{\Phi }{\mathrm{minimize}} & \sum_{i=1}^{N}{\left(P_{i}-\int_{0}^{i}PDF_{\Phi }\right)}^{2}\\ \mathrm{subject}\;\mathrm{to} & F(\Phi ). \end{array}$$

In Equation (3), Φ corresponds to the vector of parameters to be estimated. In the case of mvM, $\Phi =[\omega_{1},\kappa_{1},\mu_{1},\cdots \omega_{N},\kappa_{N},\mu_{N}]$. In the case of mWB, $\Phi =[\omega ,\alpha_{1},\alpha_{2},\beta_{1},\beta_{2}]$$PDF_{\Phi }$ is the probability density function (mvM or mWB) computed using parameters Φ. F(Φ) is a set of inequality constraints. In the case of mvM(θ), $F\left(\Phi \right)=\left[\kappa_{j}\geq 0;\;0\leq \mu_{j}<2\pi ;\;0\leq \omega_{j}\leq 1;\;\sum_{j}\omega_{j}=1\right]$. For the estimation of bWB(ν), $F\left(\Phi \right)=\left[\alpha_{1}>0;\;\alpha_{2}>0;\;\beta_{1}>0;\;\beta_{2}>0;\;0\leq \omega \leq 1\right]$. The estimation of $\Phi_{mvM}$ and $\Phi_{mWB}$ is conducted independently from each other.

*P* denotes the experimental cumulative relative frequencies (ECRF) function obtained from a sample of n independent observations. As introduced by [17,18], *P* is a discrete representation of N bins. This means that in Equation (3), least squares are computed from the difference between $P_{i}$, which is the cumulative probability from bin 0 to bin *i*, and the integral of the parametric $PDF_{\Phi }$ from 0 to bin center *i*. It is thus straightforward to integrate sensor information and airflow variability in the computation of the mixture models by using the discrete joint PMF $\Gamma_{\theta ,\nu }$. In the case of bWB(ν), *P* is equal to the marginal distribution $\Gamma_{\nu }$ while in the case of mvM(θ), *P* is equal to the marginal $\Gamma_{\theta }$.

To solve Equation (3), different optimization algorithms can be used, for example, the Levenberg – Marquardt algorithm (LMA) [24]. Solving non-linear programming problems requires, in addition, to select a starting point $\Phi_{0}$. There are several approaches to define $\Phi_{0}$, for example, [25] proposes a rule-of-thumb to determine the initial parameters for bWB(ν) while [17] proposes to use a histogram-based approach. In this work, we opted for a K-means clustering approach. In the case of bWB(ν), we use K =2 and for mvM(θ) we start with K =4 and eliminate nearly empty clusters. The parameters for each mixture component are then computed using the data points of the corresponding cluster.

### 3.2. The LT Algorithm

As an approach of spatial airflow modeling (AFM), we introduced in [19] the LT algorithm, which uses PMFs $\Gamma_{i}$ estimated at measurement locations *i* to estimate PMFs $\Gamma_{q}$ at non-visited locations *q*. The LT algorithm is an extrapolation algorithm in which airflow (and hence $\Gamma_{q}$) is modeled as the linear combination of a laminar flow component (L) and a turbulent component (T). The laminar component (L) is extrapolated using the Nadaraya–Watson [26] estimator $N_{s}$ to extrapolate on a number of samples taken from the known PMFs $\Gamma_{i}$.

The turbulent (T) component is modeled as a multimodal PMF, which is computed as a weighted linear combination of PMFs $\Gamma_{i}$. Both L and T depend on a Radial Basis Function (RBF) kernel with bandwidth $\sigma_{\mathrm{LT}}$. The final computation of $\Gamma_{q}$ is performed by weighting between the L component and the T component using a turbulence indicator ρ that considers the circular variance of T and a shape factor $\psi_{\mathrm{LT}}$.

The evaluation described in [19] showed that the LT algorithm outperforms standard extrapolation methods and is not highly sensitive to parameter selection. Also, it was demonstrated that important additional information can be extracted with the LT algorithm, for example, maps that show wind variability (“turbulence maps”). Implementation details and further information about the validation of the LT algorithm can be found in [19].

### 3.3. Frequency-Domain Airflow Characterization

As stated before, the goal of performing frequency domain characterization is to detect temporal, periodic patterns, for example, periodic patterns in the wind direction. Due to the circular nature of airflow direction, conventional time series algorithms cannot be directly applied. Thus we introduce the frequency-domain airflow modeling (f-AFM ) algorithm in this paper (Algorithm 1). Using a novelty threshold ζ as its only parameter, f-AFM identifies frequency bands in which considerable periodic wind direction patterns occur.

In summary, the f-AFM algorithm goes through three main stages. First, a correlogram [27] for the wind direction time series θ is computed (lines 1 to 3 in Algorithm 1). Then, relevant frequency components are identified (lines 4 to 11). In the last stage (line 12 and 13), the frequency bands (denoted by their upper and lower limits $f_{l}$ and $f_{h}$) are determined from the relevant frequency bands.

In statistics, correlograms are used to identify periodic patterns in time series. A correlogram C of a given signal is a vector in which each element corresponds to the autocorrelation *R* of the signal at varying time lags *ℓ* [27]. For non-circular data, *R* is computed using the Pearson correlation coefficient, which is not straightforwardly applicable to circular data. Instead, we compute *R* using the circular correlation coefficient proposed by Sengupta in [28]. *R* is computed on line 2 of Algorithm 1, where $\mu_{\theta }$ and $\mu_{\ell }$ are the circular mean of the original and the lagged time series respectively and Δ(θ,μ) computes the orthodromic (i.e., great circle) distance [29] between the mean direction and a sample θ.

In the next stage, a fast Fourier transform (FFT) is applied in order to obtain the spectrum (F) and the amplitudes (A) of the spectrum of C. To identify relevant frequency components, we use a Gaussian one-class classifier [30] as a novelty detector (line 8) over the amplitudes of the frequency components. If the probability computed by the novelty detector is larger than a threshold 0<ζ<1, we consider that a particular frequency component is relevant (lines 5 to 11). The main idea is to filter out those components that have a low energy level (i.e., amplitudes close to zero).

**Algorithm 1** Frequency-domain airflow modeling (f-AFM).
**Require:**
θ,ζ
1:**for**ℓ=0 to n **do**2: $C\left(\ell \right)=\frac{\sum_{j=1}^{n}sin\left(\Delta (\theta_{j},\mu_{\theta })\right)\cdot \sin\left(\Delta (\theta_{\ell j},\mu_{\ell })\right)}{\sqrt{\sum_{j=1}^{n}sin^{2}\left(\Delta (\theta_{j},\mu_{\theta })\right)\cdot \sum_{j=1}^{n}sin^{2}\left(\Delta (\theta_{\ell j},\mu_{\ell })\right)}}$3:
**end for**
4:
[A,F]=FFT(C)
5:
$F_{o}=\left[\right]$
6:
**for all** f∈F **do**
7: $p\left(O|f\right)=1-N\left(f|\mu_{A},\sigma_{A}\right)$8: **if** p(O|f)>ζ **then**9:  $F_{o}=F_{o}\cup f$10: **end if**11:
**end for**
12:
$\sigma_{s}=1.06\sigma_{o}{\left(n_{o}\right)}^{\left(-1/5\right)}$
13:
$\left[f_{l},f_{h}\right]=MSA\left(F_{o},\sigma_{s}\right)$
14:
**return** $f_{l},f_{h}$



To identify the upper and lower bounds of the frequency bands of the periodic patterns, we run the Mean Shift Algorithm (MSA on line 13) over the vector of relevant frequency components $F_{o}$. MSA [31] is a clustering algorithm that, compared to, e.g., K-means, does not require a-priori knowledge about the number of clusters (i.e., frequency bands). Instead, the only parameter of MSA is the kernel bandwidth $\sigma_{s}$, which can be learned from the data using the Silverman rule [32] (line 12), where $\sigma_{o}$ corresponds to the standard deviation of vector $F_{o}$ and $n_{o}$ is the number of relevant frequency components (i.e., $F_{o}$’s length). Overall, the f-AFM algorithm depends on a single hyperparameter, namely the novelty threshold ζ. In a systematic way, we found that ζ should be set to a value close to 1 (e.g., ζ≈0.99).

## 4. Quantitative Validation

In order to evaluate the algorithms introduced in the previous sections, we conducted several validation processes with simulated data and measurements acquired under controlled conditions. Quantitative validation with simulated data and in semi-controlled environments allowed us to measure the predicting capabilities of the temporal and frequency domain models against ground-truth. We opted for using these datasets due to the fact that in the target environments of this work (i.e., industrial settings) it is often hard to correlate work activity records against particular changes in the indoor airflow. Moreover, these records are not detailed enough and only provide daily production quotas and do not include particular activities conducted at specific periods of times.

### 4.1. Airflow Density Validation

We validated the airflow density models with respect to their capability for identifying dominant wind directions despite turbulent conditions. These density models are components of both, the *s*-AFM and *t*-AFM algorithms. We simulated n=2000 measurements $\hat{z}=\left(\theta ,\nu \right)$ from a flow model that has a laminar component and a turbulent component as described in related literature [20,21]. The turbulent component was given as a distribution centered at a wind speed of 0.01m/s with high standard deviation on the wind direction. This is to simulate a low speed turbulent flow with constant changes in wind direction, which is commonly observed indoors. The laminar component is given as measurements acquired with a sensor model with parameters $\sigma_{\theta }$ and $\sigma_{\nu }$. These parameters correspond to confidence intervals with respect to wind direction and speed.

In our simulated data, we considered that a percentage of the measurements (denoted by $p_{t}$) corresponded to fluctuations due to turbulence. We simulated turbulent conditions by introducing sudden, short changes in wind speed and direction.

As previously explained in Section 3.1, The wind direction $\Gamma_{\theta }$ and wind speed $\Gamma_{\nu }$ distributions depend on the confidence parameters ${\hat{\sigma }}_{\theta }$ and ${\hat{\sigma }}_{\nu }$. To test the performance and the stability of our proposed wind density estimation algorithm, we computed $\Gamma_{\theta }$ and $\Gamma_{\nu }$ using the combinations of ${\hat{\sigma }}_{\theta }=\left[5^{\circ },15^{\circ },\ldots 45^{\circ }\right]$ and ${\hat{\sigma }}_{\nu }=\left[0.03\;m/s,0.04\;m/s,\ldots ,0.07\;m/s\right]$ over dataset $\hat{z}$. As a goodness-of-fit score, we use the Cramer–Von–Mises criterion $\Delta_{n}$, which compares a cumulative density function F against an empirical density function $\hat{F}$ as follows:

(4) $$\Delta_{n}=\int_{-\infty }^{\infty }{\left(F\left(x\right)-\hat{F}\left(x\right)\right)}^{2}\omega \left(x\right)dF\left(x\right)$$

In Equation (4), ω(x) is a weighting factor. When ω(x) is set to 1, higher importance is given to the dominant wind speed and direction conditions (i.e., the center of the distribution) [22]. $\hat{F}$ corresponds to the empirical distribution while F corresponds to the ground truth distribution. In our case, we compute $\hat{F}$ from the measurement vector that includes turbulence. Since we assume that the underlying ground truth PDF is not corrupted by turbulence, F is computed from non turbulent data only. Both distributions are estimated using the sensor-aware PDF estimation algorithm intruced in Section 3. Using this validation method we aim to verify that, while turbulence is present, our proposed approach is still able to assign higher confidence to those measurements that correspond to laminar flow.

**Figure 2:**
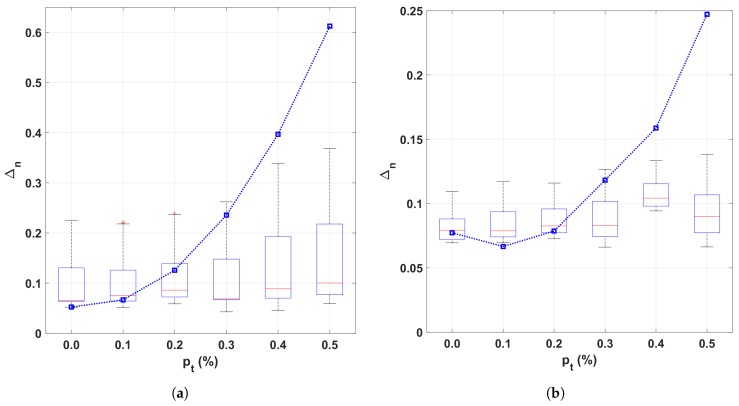
Comparison between the sensor-aware probability density function (PDF) wind models (boxplots using different values of $\hat{\sigma_{\nu }}$, and $\hat{\sigma_{\theta }}$) and standard airflow mixture models (dashed blue line). The Cramer–Von Mises criterion is used as evaluation metric). (**a**) Wind direction. (**b**) Wind speed.

Figure 2a,b show performance plots for wind speed and direction, respectively, over different amounts of “turbulent samples” $p_{t}$. Each boxplot was estimated using combinations of ${\hat{\sigma }}_{\theta }$ and ${\hat{\sigma }}_{\nu }$ and the blue dashed plot corresponds to the standard computations of mvM and mWB, which do not consider the sensor model [17,18]. As can be seen in the plots, at lower turbulence percentages both methods are comparable. However, as the turbulence percentage increases, the performance of the standard mvM and mWB estimates deteriorate exponentially while our proposed approach remains stable regardless of the selection of $\sigma_{\theta }$, $\sigma_{\nu }$ and the noise percentage $p_{t}$.

### 4.2. *s*-AFM Algorithm Validation

As mentioned above, the spatial component is computed by the *stf*-AFM algorithm using the LT algorithm originally presented in [19]. A detailed description of the validation process can be found on the original publication. In this work we present a brief summary. The quantitative validation was conducted with real world datasets acquired with different mobile robotic platforms and the goal of the validation process was: (1) to compare the prediction capabilities of the LT algorithm against conventional extrapolation techniques; (2) to determine the sensitivity of the algorithm to the selection of its meta-parameters and (3) to explore the capabilities of the LT algorithm to identify temporal patterns that remain over long periods of time.

Regarding (1) and (2), leave-one-out cross validation (LOOCV) was conducted with airflow data acquired with an unmanned aerial vehicle that explored an outdoor environment following a pre-defined path. In 11 measurement tours conducted over several days, the UAV stopped to measure airflow at pre-defined way-points. For each run, LOOCV was conducted leaving one way-point out for testing. In total, 15,000 models were trained and tested using different parameters for the LT algorithm. One important conclusion of this extensive validation process was that the LT algorithm outperforms conventional extrapolation techniques regardless of the parameter selection.

Regarding (3), the stability of the results over time were measured using a dataset collected with a ground robot inside a foundry hall. The dataset included short-term measurement tours conducted at two different seasons in the year. A stability map, which measures the variability of the airflow maps over the different runs was computed. This stability map showed that, while local and short-term variability might be present, there are dominant airflow conditions that remain stable over the whole year.

### 4.3. *f*-AFM Algorithm Validation

We validated the f-AFM algorithm using simulated datasets and data collected with an airflow sensor (i.e., an ultrasonic anemometer) in a semi-controlled environment.

**Figure 3:**
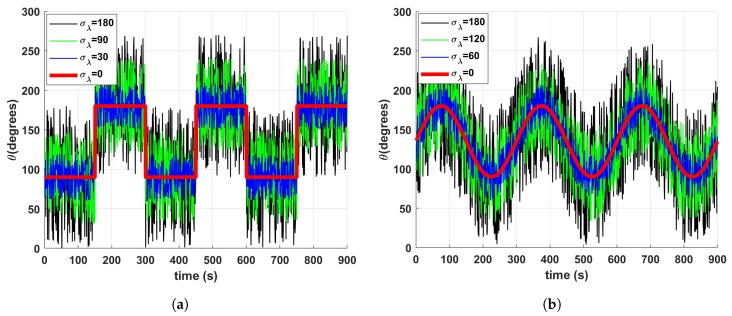
Examples of simulated wind direction signals under different noise levels. (**a**) Squared waveform. (**b**) Sinusoidal waveform.

Simulated data were generated as wind flow that periodically changes its direction according to two types of signals, namely a square and a sinusoid signal. In both cases, the wind direction oscillated between $90^{\circ }$ and $180^{\circ }$. Different data sets were generated in which we included zero mean additive noise with different standard deviation values $\sigma_{\lambda }=\left[0^{\circ },30^{\circ },60^{\circ },\ldots ,180^{\circ }\right]$. In all the simulations, the period of the signals and the total duration of experiments was kept constant to T = 300 s and 3000 s, respectively. We generated 20 datasets for each value of $\sigma_{\lambda }$ for a total of 280 simulations considering both waveforms. Figure 3a,b show examples of different simulated time series.

**Figure 4:**
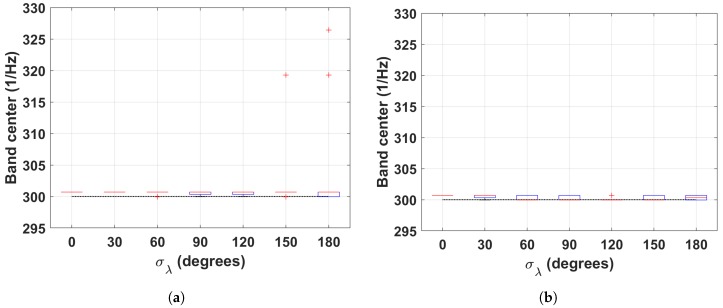
Results summary of the f−AFM algorithm. Notice that the median highest energy spectral density HESD (red lines) closely follow the period of the simulated signals (dahsed lines). (**a**) Squared signal. (**b**) Sinusoidal signal.

To evaluate the capabilities of the f-AFM algorithm to locate relevant frequency bands, we select the band that has the highest energy spectral density (HESD), and we compute its center. Figure 4a,b summarize the validation results with the simulated dataset. In the figures, we computed boxplots for the different values of $\sigma_{\lambda }$. The dashed black line denotes the ground truth period (300s). Notice that the median of the estimated HESD band centers does not deviate considerably from the ground truth. Furthermore, the predictions do not vary considerably, as can be seen by the width of the inter-quartile-range and the location and number of outliers.

In a second validation step, we collected data in a semi-controlled environment. The experimental setup consisted of a fan placed at a different distances d=[0.20,0.50,1.00,1.50,2.00,4.00]m from an anemometer. For d=0.20m to d=2.00m, no obstruction was placed between the anemometer and the fan. In the case of d=4.00m, the anemometer and the fan were placed at opposite ends of a busy room. Between them, people walked by or sat nearby to carry out different activities. The fan performed a panning movement (left-center-right-center) with a period of T≈13m. The anemometer was an FT702-LM (https://www.fttechnologies.com/uploads/files/1/Resource/FT702LM-Data-Sheet-ENG.pdf) from the manufacturer FT technologies Ltd, Teddington, UK. This anemometer measures wind with a resolution $1^{\circ }$ and offers an accuracy of $\pm 4^{\circ }$.

**Figure 5:**
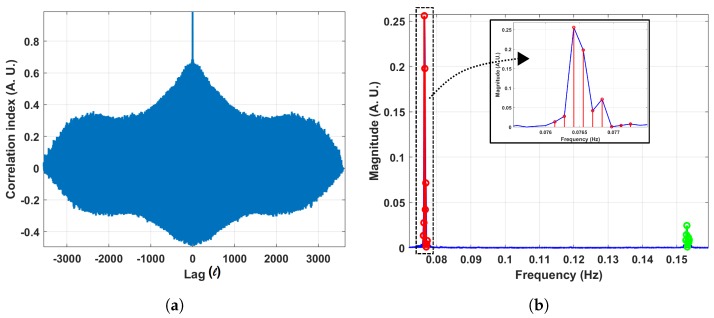
Results for the experimental run where the distance *d* between the fan and the anemometer was 0.50m. (**a**) Correlogram; (**b**) Frequency spectrum where the HESD is highlighted in red.

The results of the second step validation tests are summarized in Table 1 and an example of a correlogram and the corresponding frequency spectrum is shown in Figure 5a,b. As with the simulated data, we extracted the HESD band. We also list the number of bands detected. Please notice that in all trials, the fan dominated the airflow as indicated by the HESD (in which the periodic frequency of the panning fan’s motion is situated). It can also be noticed that, as the distance to the fan *d* increased, more bands were detected. This is an expected result since the influence of the fan should decrease with distance to the anemometer. In the case of d=4.00 m a considerable number of bands was extracted. These additional spectral bands, however, have a low energy and can be related to motions that disturb the airflow.

## 5. Measurement System

**Figure 6:**
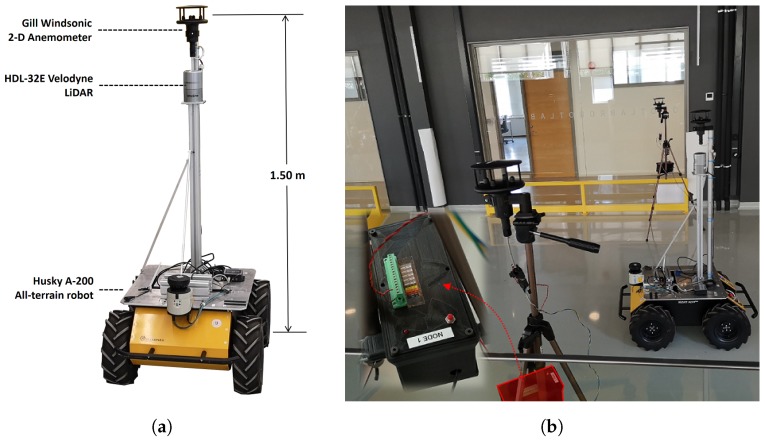
Measurement system used in this paper. (**a**) Mobile robot. (**b**) Sensing nodes.

The measurement system we developed comprises a mobile robot equipped with localization and airflow sensing modalities and a set of five robust sensing nodes. The robotic platform (Figure 6a), is a Husky A-200 (http://www.clearpathrobotics.com/husky/) equipped with an HDL-32E 3D LiDAR used to localize the robot. The HDL-32E allowed us to generate point clouds of up to 700,000 points per second with a 100 m range and an accuracy of ±0.02 m at 10 Hz. To assure robust robot localization, which is critical to associate measurements with spatial locations, we employed the normal distribution transform Monte Carlo localization (NDT-MCL) in large, 3D environments [33].

For airflow sensing, the robot was equipped with a Gill Windsonic anemometer (http://gillinstruments.com/products/anemometer/windsonic.htm) on top of a 1.5 m metal pole. The Windsonic anemometer is a robust, low cost 2D ultrasonic wind sensor with no moving parts. According to its manufacturers, it has a measurement range of 0–60 m/s with a wind speed accuracy of ±2% at 12 m/s (at a resolution of 0.01 m/s) and a wind direction accuracy of $\pm 2^{\circ }$ at 12 m/s (at a resolution of $1^{\circ }$). We collected wind data with the robot at a sampling frequency of 1 Hz.

We built five robust sensing nodes (Figure 6b) that at their core contain an Arduino (MEGA 2560) microcontroller board. The sensing nodes can collect data from RS232 sensors (e.g., the Gill Windsonic anemometer), from analog inputs and serial devices (i.e., I2C) such as temperature and humidity sensors. Measurements are stored internally or transmitted via WiFi to a remote server. As on the robot, a Gill Windsonic anemometer was used in the sensing nodes to collect wind data at a 1 Hz sampling rate.

The sensing nodes were developed in such a way that they can operate continuously largely irrespective of the environmental conditions. The microcontroller board is enclosed in a 3D printed case which protects from dust, smoke, high temperatures and fluids. The sensing nodes include a real-time clock that keeps the current time and date. We added synchronization capabilities with an external server in order to prevent drift of the internal clock.

## 6. System Deployment

In order to explore the applicability of the proposed system in real world applications, we addressed the problem of ventilation characterization in two different industrial scenarios. Note that due to the companies’ secrecy policy we cannot include in this paper labels of the different locations in the work space or information about the production process that was learned by the ventilation characterization approach presented in this paper.

The first industrial environment we considered is a foundry that casts bronze bearings. In this scenario, several activities are conducted inside a 12,800 m${}^{3}$ area. For example, bronze scrap is melted and then transported to locations where bearings are being cast. We refer to this scenario as foundry-A. During data collection, personnel conducted their assigned tasks as usual and thus, no disruption or artificial conditions were introduced. Moreover, the ventilation system (which uses a mixing principle) of the facility was operating according to the schedule defined by the foundry’s operators.

The second industrial scenario we surveyed is a foundry that specializes in the production of high volume cast products (referred to in the following as foundry-B). In foundry-B several processes that generate high volumes of airborne by-products are carried out inside a 44,350 m${}^{3}$ space. On a typical day, ore is transported to one of the smelter furnaces. Concurrently, moulds are created, coated and let to rest before pouring metal into them. After the process is done, the recently cast goods are let to rest for some time. These processes generate a significant amount of by-product gases and dust pollution. To counteract this situation, plant operators use a displacement ventilation approach. Which means that pollutants are transported outside the work space by using large gates that are open or close depending on the activity being conducted in the work space.

### 6.1. Ventilation Characterization, Foundry-A

In foundry-A, we collected data with 5 sensing nodes placed at different positions as indicated by the square markers in Figure 10a,b. There was no automatic heuristic/sensor planning involved in the positioning of the sensors. The final locations were decided as a compromise between the demand for area coverage, the availability of power connectors and the aim to minimize disturbance of the foundry’s processes. As shown in Figure 10a, the sensor nodes are labeled S-1 to S-5. Due to the particular work processes in foundry-A, specific activities are being carried out at different positions during the working day. In total, data samples were collected during 11 days. Since one wind sample was acquired every four seconds, we collected approx. 1,080,000 samples with the sensing nodes in total. Additionally, we collected data with the mobile robot by performing different exploration tours (indicated as dashed white lines in the maps shown below). Each of these tours lasted 30–100 min during which the robot stopped on average approx. 5 min to collect measurements. In total, 190 positions (white markers in the maps) were sampled with the robot.

**Figure 7:**
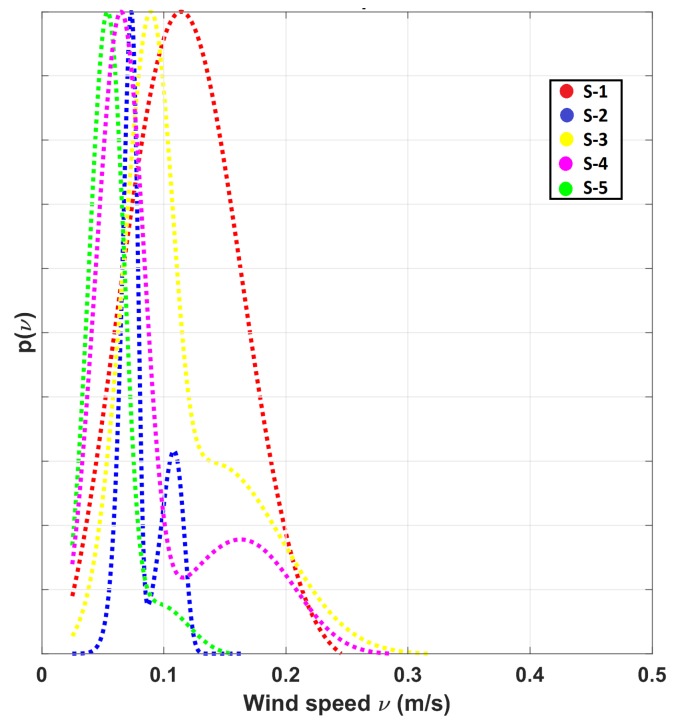
Bi-modal Weibull distributions bWB(ν) computed at the different locations of the sensing nodes in foundry-A.

Figure 7 shows wind speed distribution models bWB(ν) for each of the locations measured with the sensing nodes. These distributions were computed during working days (Monday to Friday) when the ventilation system is operating. Notice that all of them are rather narrow distributions with similar expected means. In foundry-A we did not observe large variations of the wind speed between the sensor nodes.

In order to get an idea about how the airflow distribution varies over time, we estimated ventilation calendars, which form a time-dependent model of the airflow at a given measurement position. These calendars show expected wind speed, wind direction and the turbulence indicator for a given period in time. While in the calendars only expected values are shown, each cell is associated to full PDFs over wind speed and direction (bWB(ν) and mvM(θ)). The turbulence calendar measures the stability of airflow as a value between 0 and 1. The stability index corresponds to the inverse of the circular variance (CV), which is the angular variation around a mean direction. The CV is bounded between 0 and 1, reaching its maximum when wind vectors vary between opposing directions [34].

**Figure 8:**
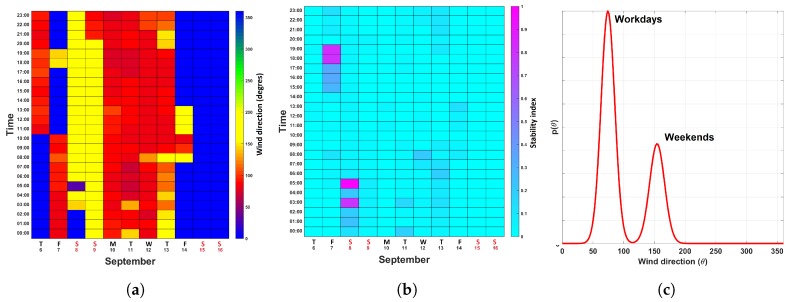
Ventilation calendars for sensor location S-1. (**a**) Wind direction calendar. (**b**) Turbulence calendar. (**c**) mvM(θ) computed from data collected from September 6th to September 16th.

**Figure 9:**
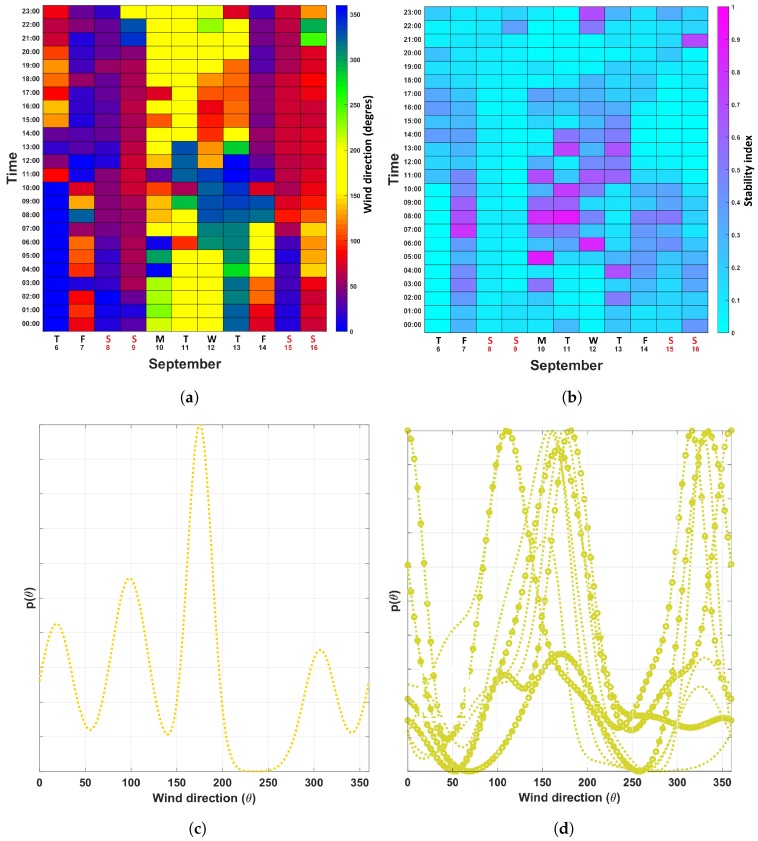
Ventilation calendars for location S-3. (**a**) Wind direction calendar. (**b**) Turbulence calendar. (**c**) Global mvM(θ) calendar. (**d**) Hourly distributions mvM(θ) computed with data collected during the morning of September 10th and September 11th, 2018.

Figure 8a,b and Figure 9a,b show the ventilation calendars for the two sensing nodes *S-1* and S-3. The airflow at S-1 was found to be rather stable, as can be seen in the wind direction calendar (Figure 8a) and the turbulence calendar (Figure 8b). Please notice that the wind direction noticeably changed on Friday, September 7th. This is because the ventilation is being turned off when no activities are being carried out in the foundry. This change in the wind direction is shown in Figure 8c, where mvM(θ) for the workdays and the weekends are displayed.

Figure 9a–d correspond to sensing node S-3. Compared to node S-1, we observed a higher degree of variability. This is due to the fact that S-3 is located in an area where, for example, metal ore and finished goods are transported, and extractors are activated/deactivated periodically. Nevertheless, we can observe clear patterns as shown by the multimodal distribution mvM(θ) shown in Figure 9c. Here, each of the modes corresponds to a particular day where a set of activities were conducted. Interestingly the turbulence map (Figure 9b) shows a rather stable behaviour, with the exception being the early mornings of Monday and Tuesday, 10 and 11 September 2018. During these times the turbulence indicators were close to 1. Zooming into these particular time spans (Figure 9d), we observed indeed, that the distribution mvM(θ)s for these days comprise multiple modes in opposite directions.

**Figure 10:**
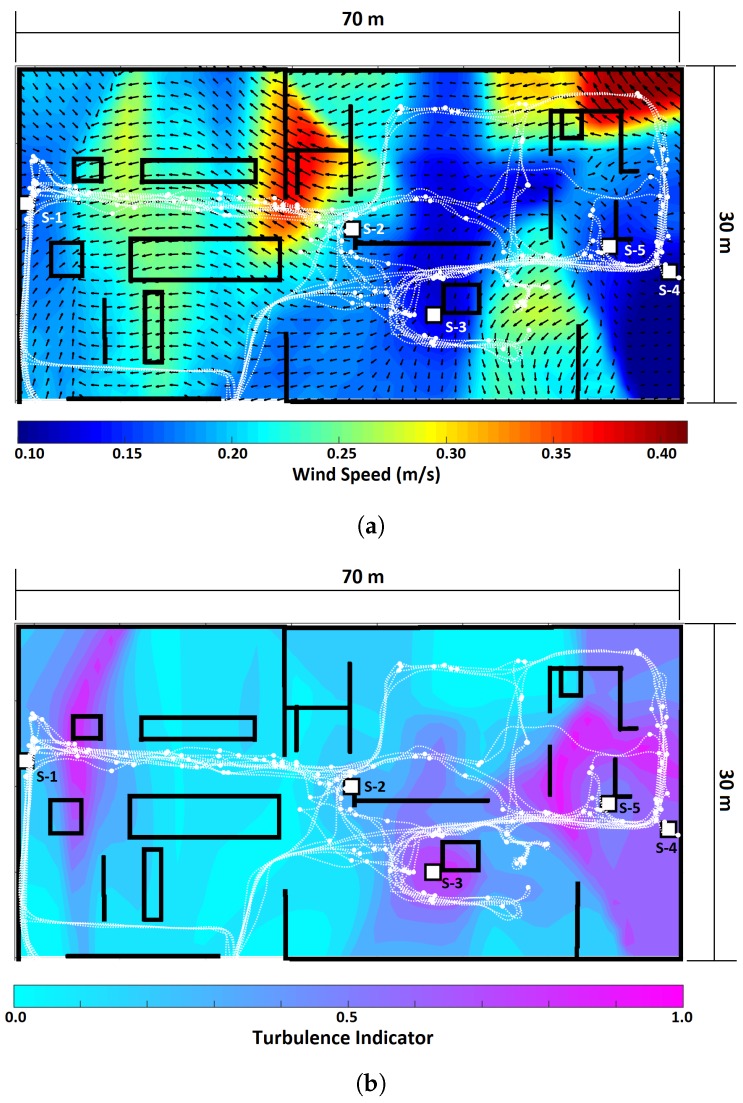
Ventilation maps for foundry-A. (**a**) Expected wind map, where the arrows indicate the wind direction while the color code denotes the wind speed. (**b**) Turbulence map.

We also estimated a ventilation map for foundry-A, i.e., a snapshot of the airflow conditions computed from localized measurements at *n* way points. To compute the ventilation map, we used the data collected during all 12 robot exploration tours and the data from the sensing nodes while exploration was being conducted. Figure 10a,b show the expected wind and turbulence map. In the wind map, the expected wind directions are denoted by arrows, and the wind speed is color coded, see Figure 10a. For each of the locations in the map, we estimate associated distributions mWB(ν) and mvM(θ). Due to the variability of the airflow conditions and lack of ground truth, it is hard to analyze these maps quantitatively. However, the ventilation maps and the ventilation calendars were analyzed with the help of the ventilation expert of foundry-A. The expert commented that the results match the expected behaviour of the airflow. The experts pointed out that it is particularly interesting to observe that the direction of the airflow keeps the contaminants enclosed around location 1 (where S-1 was placed), which is the area where high volumes of dust, respirable matter and gases are released. In addition, location 3 (near S-3) was predicted to be highly turbulent, which matches the behaviour observed in the ventilation calendar. The ventilation expert also stated that the behaviour at location 4 (where S-4 and S-5 were placed) does not fully match the expected behaviour. We believe that the maps in this area do not represent the actual airflow very well due to two possible reasons: first, the position of the S-4 and S-5 was less than optimal due to safety restrictions. Second, it is likely that more sensors are needed in order to have a better coverage of location 4. Optimizing sensor placement (i.e., finding unobstructed, informative positions) is indeed an interesting line of research. However, it is out of the scope of this work.

### 6.2. Ventilation Characterization, *Foundry-B*

In foundry-B, five sensing nodes where deployed at different locations in the working space. The data collection process lasted from 18–25 October 2018. Due to time and resource constraints, no data were collected with the robot.

**Figure 11:**
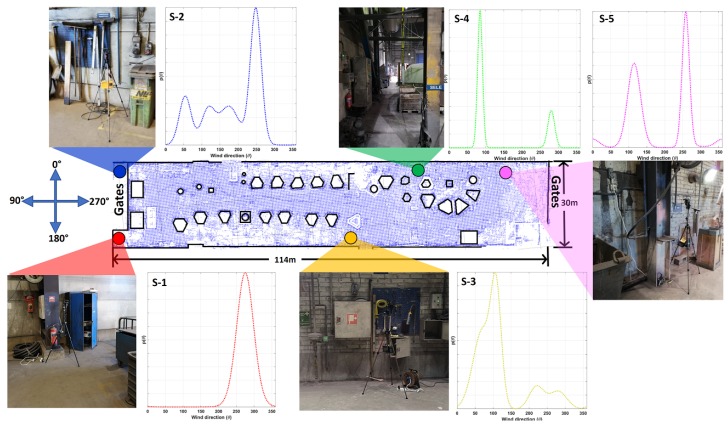
Summary of the characterization performed at foundry-B. At each sensor location S-1 to S-5 models mvM(θ) are shown.

As previously stated, foundry-B uses a displacement principle for ventilation and thus, the behaviour of the airflow is highly dependent on the status of the air intake mechanism, which in the case of foundry-B consists of two 5 m wide gates that are opened when casting is being performed. In addition to their ventilation function, these gates are also opened when raw material arrives to the facility to be melted in the furnace. The second set of gates is located at the other end of the facility. Figure 11, shows the layout of the work space and wind direction distributions $\Gamma_{\theta }$ computed from the full data set collected over the full measurement campaign during work days (We obtained a full data set with one restriction. The internal memory of node S-1 malfunctioned after two days of operation, and we could only use the measurements up until then).

At S-2 we observed a high wind direction variability expressed by a multimodal distribution. However, a dominant wind direction could still be observed, which suggest that the airflow at location S-2 is likely to flow from the gates towards the work space.

**Figure 12:**
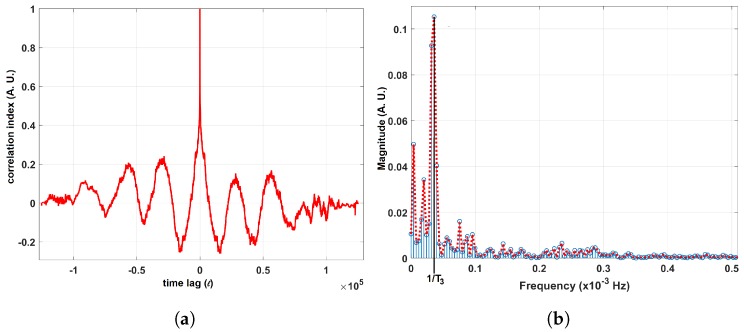
(**a**) Correlogram computed from data collected with sensor S-5 during October 18th and October 19th. (**b**) The corresponding frequency spectrum.

In addition, we conducted the frequency analysis described above to determine whether or not there were specific temporal patterns in the wind direction fluctuations at each node. In order to do so, we carried out the f-AFM procedure to each of the time series acquired with the sensing nodes. We observed several high energy frequency components, in particular three components with periods $T_{1}$, $T_{2}$ and $T_{3}$ (Due to the foundry’s secrecy policy, we cannot state the actual values for $T_{1}$, $T_{2}$ and $T_{3}$ in this paper.) during different days of the measurement campaign. The foundry operators confirmed that $T_{1}$, $T_{2}$ and $T_{3}$ are correlated with periodic activities conducted during the manufacturing process. An example of these results is shown in Figure 12a,b, that show the frequency domain analysis conducted over data collected on 18 and 19 October at location S-5. It can be seen in the plot of the auto-correlation over the wind direction in Figure 12a) that the correlogram reaches its maximum with a time lag ℓ=0. In addition, local maxima appear periodically. In the frequency spectrum (Figure 12b) this corresponds to a high energy component located at frequency $\frac{1}{T_{3}}$.

## 7. Conclusions

In this paper, we presented a comprehensive approach for airflow modelling (AFM) targeted for micro-scales, in particular, indoor environments. Micro-scale, indoor AFM is a task that has direct applications in different research areas and industries, including environmental monitoring, mobile robot olfaction and occupational health. The algorithms we presented in this paper perform AFM in three different domains, namely in the temporal, spatial and frequency domain. For both, the temporal and spatial domain, we used mixture models to estimate continuous PDFs of wind direction and wind speed. To identify dominant wind directions, our modeling technique incorporates knowledge about the sensors and knowledge about the variability of the wind flow. With respect to spatial modeling, the LT algorithm is used to estimate PDFs at non-measurement locations. We further developed our AFM approach by introducing the f-AFM algorithm to characterize airflow in the frequency domain. The f-AFM algorithm allows to identify frequency bands in which substantial periodic wind direction patterns are observed. We validated our algorithms in simulated and semi-controlled environments where we demonstrated that the proposed algorithms outperform conventional PDF estimation techniques and that they can identify relevant frequency bands in controlled and semi-controlled scenarios.

We showed the applicability of our algorithms and the importance of the task of AFM by deploying the system at two different foundry sites. From the discussion with foundry operators, we verified that the task of AFM to characterize ventilation systems is of high interest in the industry. The main advantage of ventilation characterization from the perspective of foundry operators is to improve occupational health conditions by better understanding indoor airflow. In particular, the models we presented to the foundry operators allowed them to verify where their ventilation system is operating according to their expectations and where this is not the case. We obtained particularly interesting results with the f-AFM algorithm which, regardless of the uncontrolled nature of the surveyed environment, was able to identify the periodicity of the work events that dominate the airflow inside the foundry.

The results presented in this paper are encouraging and open several opportunities for further research efforts and future work. For example, further evaluation should be conducted to study the effect that the discretization step has in the computation of the PMFs and the PDFs. In our current implementation, we only consider bi-variate Weibull distributions. Future work could consider the use of multimodal Weibull distributions as well. For modelling the distribution over wind directions, we use a mixture of Von–Mises distributions with a pre-defined number of modes and a pruning step in which we eliminate empty clusters. Here, an improved algorithm that automatically determines the number of modes could be developed, similar to the work presented in [35]. Another line for future work is to improve the spatial model. Currently, the LT algorithm computes a snapshot of the airflow only when the robot is present. An algorithm that learns the extrapolation model (such as the one presented in [2]) could be adapted for the specific task of airflow modelling and ventilation characterization.

## Figures and Tables

**Table 1:** Evaluation of the *f*-airflow modeling (AFM) algorithm in semi-controlled environments, summary of results.

Distance (m)	HESD (1/Hz)	Bands Detected
0.20	[12.80,13.13]	3
0.50	[13.01,13.14]	3
1.00	[12.88,13.09]	3
1.50	[12.88,13.17]	3
2.00	[12.91,14.86]	4
4.00	[11.09,14.14]	13
